# Acupuncture on Management of Post‐Traumatic Trigeminal Neuropathic Pain: A 24‐Month Follow‐Up Case Report

**DOI:** 10.1111/scd.70094

**Published:** 2025-09-01

**Authors:** Lucas Masaru Marubayashi, Mtheus Herreira Ferreira, Carolina Paes Torres, Rodrigo Galo, Maria Cristina Borsatto

**Affiliations:** ^1^ Department of Pediatric Dentistry, Faculty of Dentistry of Ribeirao Preto University of São Paulo Ribeirão Preto Brazil; ^2^ Department of Biological Sciences, Bauru School of Dentistry University of São Paulo Bauru Brazil; ^3^ Department of Dental Materials and Prosthesis, Faculty of Dentistry of Ribeirao Preto University of São Paulo Ribeirão Preto Brazil

**Keywords:** acupuncture, somatosensory disorders, trigeminal nerve injuries

## Abstract

Neuropathic pain and its high impact on patient's quality of life, must be thoroughly assessed to be adequately managed. Unfortunately, there remains a lack of evidence for different treatment options that could improve the quality of life of those patients. Beyond first‐choice therapies, complementary integrative therapies can emerge as a highly valuable option for controlling chronic pain. Thus, the aim of this study was to report a case of acupuncture utilized to manage chronic neuropathic pain associated with sensory loss following trauma after dental implant surgery. A 41‐year‐old female patient presented with a complaint of left‐sided intraoral pain (visual analogic scale [VAS] = 9) in the posterior mandibula region over the last year following a dental implant surgery. On physical examination, the patient exhibited hypoesthesia in the region identified by qualitative somatosensory tests. The implant showed no clinical or tomographic alterations. The initial diagnosis was probable post‐traumatic neuropathic pain (PTNP). As the patient opted against initiating central‐acting medications, five sessions of acupuncture were performed. Following the proposed treatment, there was a significant reduction in the patient's pain report (VAS = 1) and a significant decrease in the sensory loss area. We can therefore conclude, that in this case, the use of acupuncture was effective in controlling pain and significantly reducing the sensory loss area caused by PTNP.

## 1 Introduction

Neuropathic pain—defined by the International Association for Study of Pain (IASP) as a pain that arises as a direct consequence of a lesion or diseases affecting the somatosensory system—is a complex and debilitating condition that arises from damage or dysfunction within the nervous system, whether peripheral or central [1, 2, 3, 4, 5]. Its onset can be caused by injury followed by inflammation [4] sustained by mediators from affected C‐fibers, reported as burning sensations, tingling, and/or altered sensitivity to touch or pressure stimuli [3].

Several dental and maxillofacial surgery procedures can lead to post‐traumatic neuropathies [1, 3]. The increase in dental procedures may be associated with a rise in the occurrence of injuries to the trigeminal nerve, which can result in neuropathies ranging from loss of sensitivity to severe pain [3], severely diminishing patients’ quality of life [2, 4, 6].

The management of neuropathic pain represents a significant challenge for both patients and healthcare professionals. Medications often provide limited relief and can be accompanied by undesirable side effects [7]. Recently, medical research has explored alternative and complementary therapies to treat neuropathic pain, among which acupuncture has emerged as a promising candidate [6]. Although its mechanism of action is complex and remains unclear, acupuncture acts at various levels, locally and systemically, modulating motor, sensory, autonomic, neuroendocrine, and emotional functions [8]. It appears to be an interesting alternative in cases of chronic pain.

Therefore, the aim of this study was to report a case of chronic neuropathic pain, along with electric shocks sensation and sensory loss, following dental implant surgery in the mandible, managed with acupuncture treatment.

### 1.1 Case Report

The present case was written in accordance with CARE guidelines [9]. A 41‐year‐old female patient (L.G.B.) was referred to the specialized acupuncture service at the Ribeirão Preto School of Dentistry (FORP‐USP), reporting left‐sided intraoral pain over the past year. This pain initiated after a dental implant surgery in the edentulous region of the first left inferior molar. The patient reported experiencing an electric shock sensation at the time of the anesthetic injection during the inferior alveolar nerve block. The same painful, shock‐like sensations continued throughout the surgery and persisted postoperatively. The patient stated alerting the professional in charge of the surgery, who nevertheless chose to proceed with the procedure. After the intervention, the patient experienced the same symptoms for the last year (since the procedure was performed): constant electric shock‐like pain on the left side of the face, in the posterior mandibular region, both intra‐ and extra‐orally, accompanied by a sensation of touch sensory loss. The patient reported no general health comorbidities and no family history of similar condition. Also reported no significant psychosocial impairment or any pain in other body areas. Aside from the painful condition, the patient reported no other abnormalities in the postoperative period and indicated that no intervention had been performed to control the present symptoms until this moment.

**FIGURE 1:**
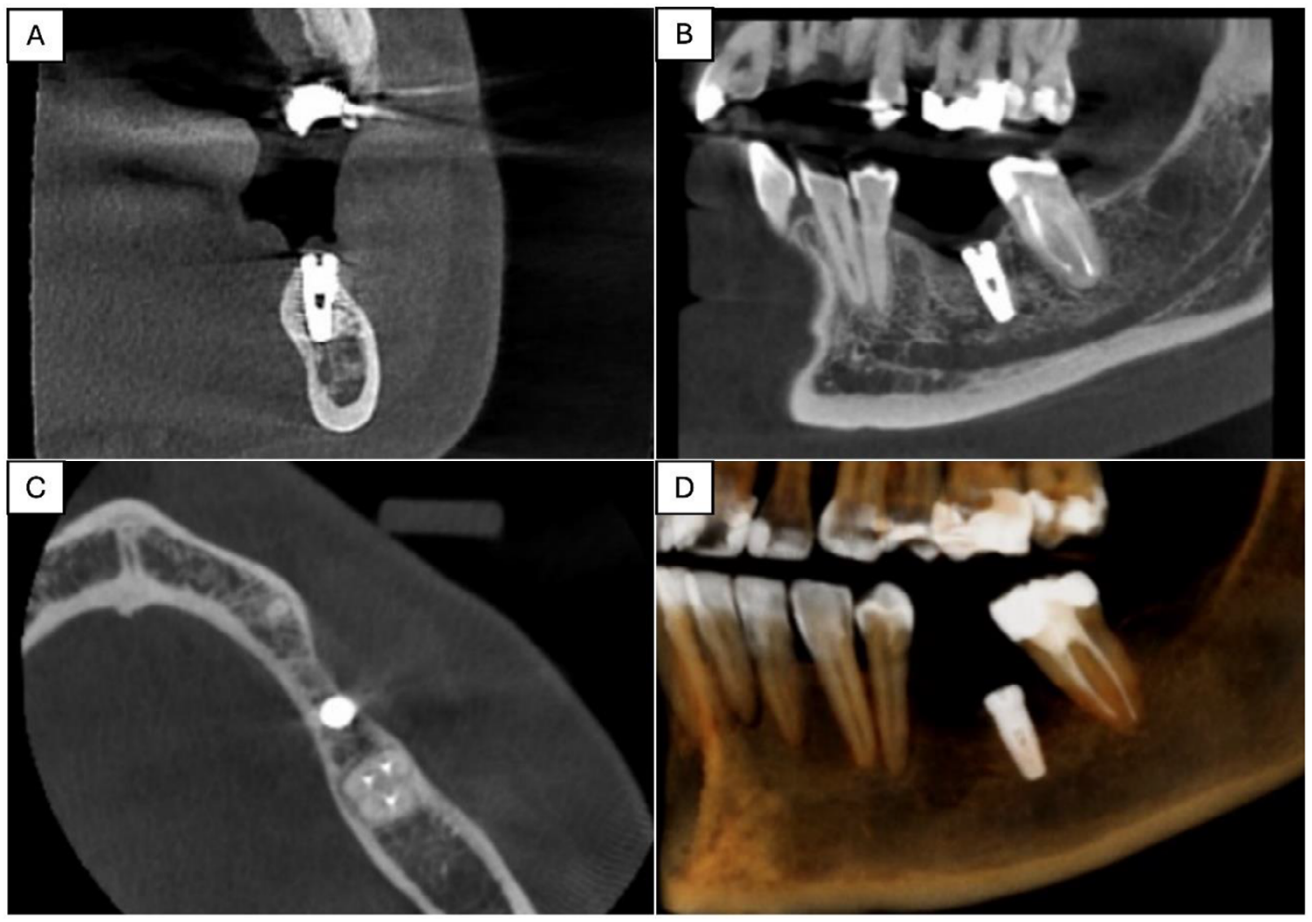
Cone beam computed tomography (CBCT) dental implant scan: (A) Coronal plane; (B) Sagittal plane; (C) Axial plane; and (D) 3D reformatting.

Clinical examination revealed the presence of the edentulous space where the implant had been installed, with no clinical alterations other than hypoesthesia to touch. To better visualize the implant, which was covered by local gingival tissue and lacked an implant prosthesis, a cone beam computed tomography (CBCT) scan was requested. The CBCT scan showed that the dental implant was in a good position, respecting the limits of the mandibular canal and with imaging signs compatible with osseointegration (Figure 1).

**FIGURE 2:**
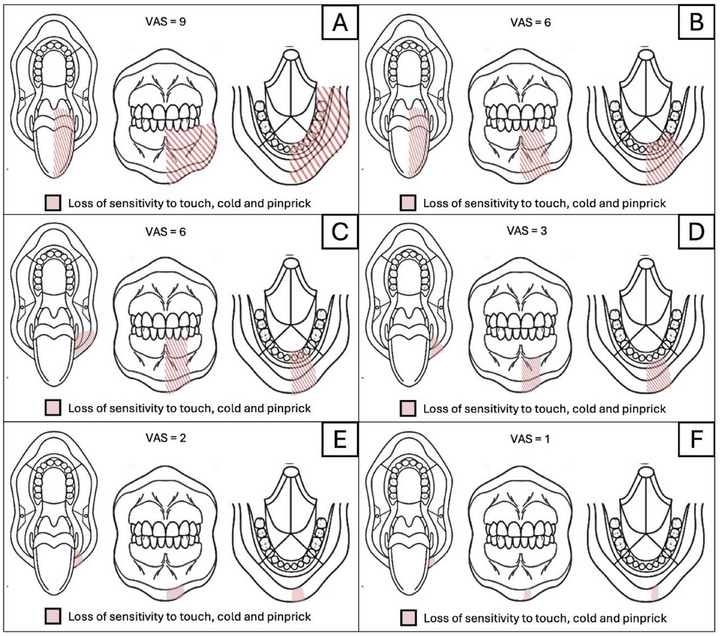
Intraoral Qualitative Sensory Testing (QualST) and Visual Analogic Scale (VAS) values in the following times: (A) Initial assessment (Before first acupuncture session); (B) Second acupuncture session (1‐week follow‐up); (C) Third acupuncture session (2‐week follow‐up); (D) Fourth acupuncture session (3‐week follow‐up); (E) Fourth acupuncture session (4‐week follow‐up); and **(F)** Fifth acupuncture session (5‐week follow‐up).

To better assess the patient's condition, we decided to carry out the Qualitative Sensory Test (QualST) [10, 11] to map the condition and control follow‐up. The test revealed hypoesthesia to touch, cold, and pinprick when compared to the unaffected side, extending from the lower left central incisor to the ascending mandibular ramus, in the buccal and lingual intraoral mucosa, and in the skin of the same region (Figure 2A). The patient's pain level was also measured using the 10 cm visual analog scale (VAS) [12], which was converted to a numerical scale rating (NRS), where the patient reported a severe pain (VAS = 9). Other tests to rule out pain of odontogenic origin were performed, yielding negative results.

After extensive evaluation and compilation of clinical information and the patient's report, according to the International Classification of Orofacial Pain (ICOP) criteria [13], the final diagnosis was probable post‐traumatic trigeminal neuropathic pain (PTNP). This diagnosis was reached due to the anatomical normality seen in patient's CBCT, coupled with the absence of other tests that could confirm the lesion, and the signs and symptoms reported by the patient. Given this condition and the patient's refusal to use first‐choice medications, we opted for integrative therapy with acupuncture to control the condition.

**FIGURE 3:**
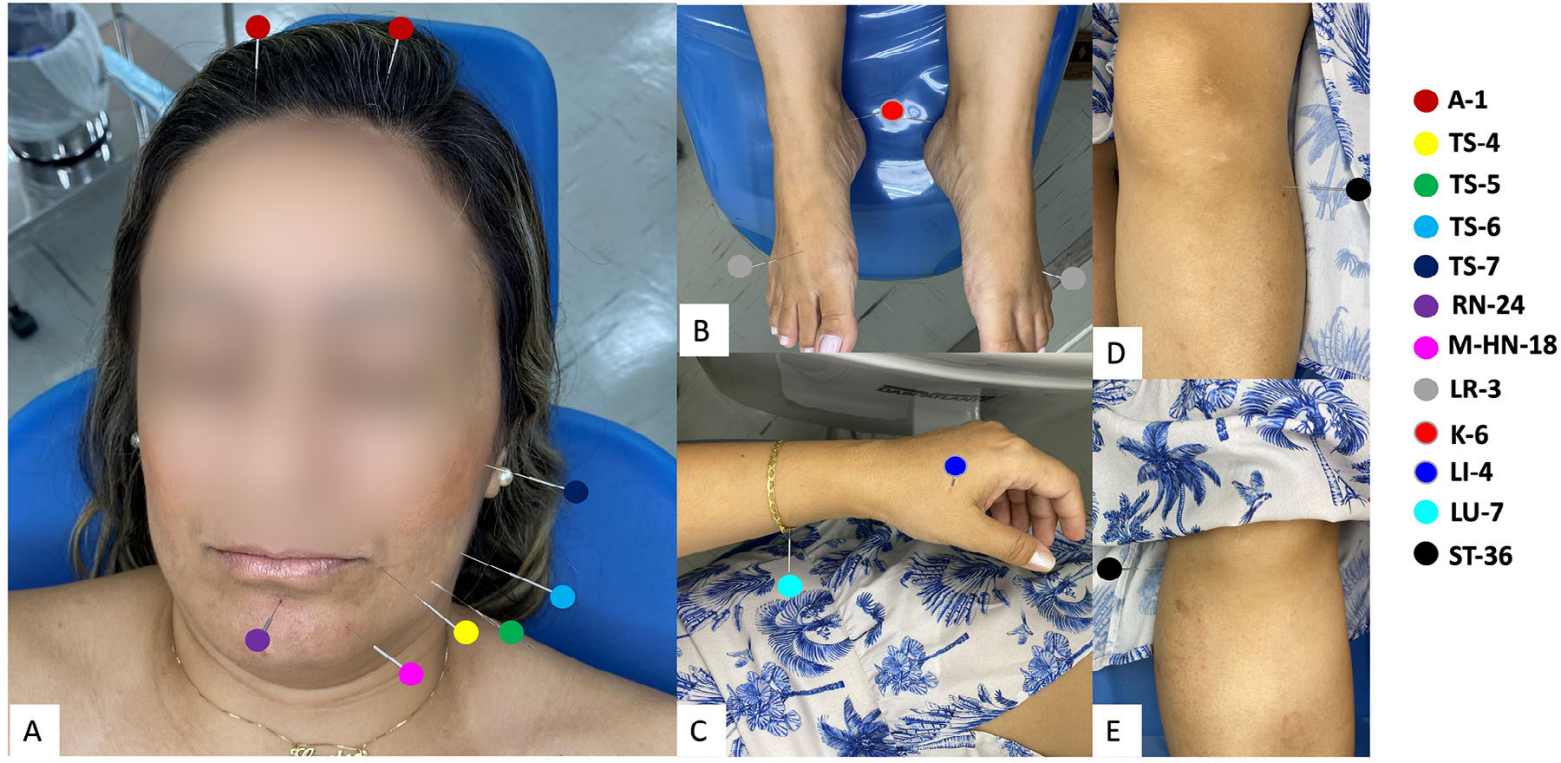
Elected puncture points: (A) Facial points (A‐1 – Ynsa, ST‐4 – Dicang, ST‐5 – Daying, ST‐6 – Jiache, TS‐7 – Xiaguan, RN‐24 – Chengjiang and M‐HN‐18 – Jiachengjiang; (B) Feet points (K‐6 – Zhaohai and LR‐3 – Taichong); (C) Hand points (LU‐7 – Lieque‐point and LI‐4 – Hegu); (D) and (E) Leg points (ST‐36 – ZuSanLi).

The patient underwent a total of five acupuncture sessions, with one session per week for a total of 5 weeks. In each session, the QualST test was initially carried out, and the VAS was measured to monitor the condition (Figure 2A–F). Subsequently, the established protocol for this case was applied involving the puncture of points LU‐7 (Lieque‐point), LR‐3 (Taichong), ST‐36 (ZuSanLi), LI‐4 (Hegu), A1 (Ynsa), ST‐4 (Dicang), ST‐5 (Daying), ST‐6 (Jiache), ST7 (Xiaguan), M‐HN‐18 (Jiachengjiang) RN‐24 (Chengjiang), and K‐6 (Zhaohai), which are points related to the patient's affected region (Figure 3). After the sections were completed, the patient was followed up at 1, 2, 3, 6, 12, and 24 months.

**FIGURE 4:**
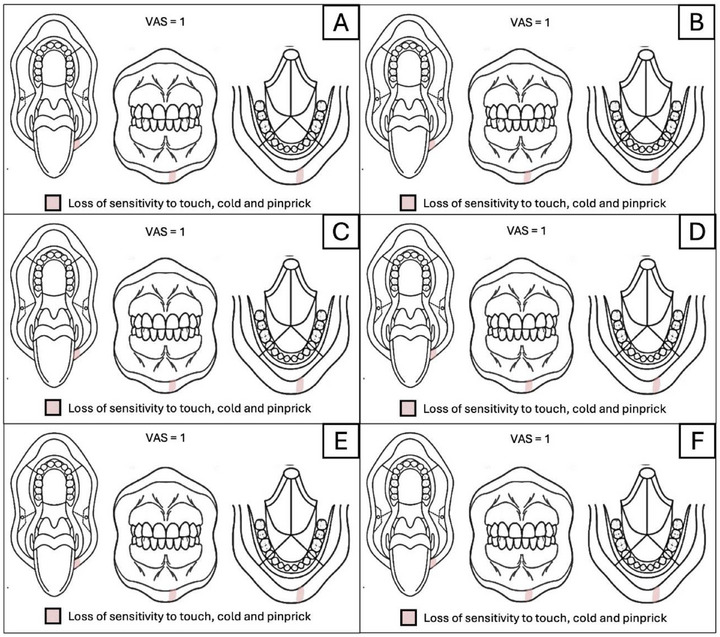
Intraoral Qualitative Sensory Testing (QualST) and Visual Analogic Scale (VAS) values follow‐ups: (A) 1 month; (B) 2 months; (C) 3 months; (D) 6 months; (E) 12 months; (F) 24 months.

The sequence of assessments and the evolution of the patient's signs and symptoms can be seen in Figure 4. The patient made significant progress both in terms of reported pain and in the areas of hypoesthesia detected during the initial assessment. The initial VAS of 9 on the affected side was reduced to 1 in the fifth and final acupuncture session, and the area of hypofunction was limited to just a small area on the lower lip (Figure 2F). The patient reported that “she could feel her teeth again when eating” and now experienced “minimal discomfort”, which rarely increases in intensity and or in area of hypoesthesia during daily stress events. The patient is currently being followed up, with the same pain and sensory loss persisting for a period of 24 months (March 2023–March 2025) (Figure 4), with no reported adverse effects report and good tolerability. The informed consent for this case report was obtained from the patient.

## 2 Discussion

In this paper, we discuss the use of acupuncture for management of probable trigeminal neuropathic pain. For this condition, the use of centrally acting medications has become the treatment of first choice, as elucidated in the literature. However, in view of the patient's refusal of pharmacological treatment, the use of acupuncture may be a promising alternative [6].

PTNP is the result of a complex pathophysiological mechanism involving alterations in the central and peripheral nervous systems [3, 4, 11], which make its diagnosis and control challenging. Injury or damage to the trigeminal nerve triggers a series of adaptive responses leading to an exacerbated perception of pain [3, 11, 14]. Subsequent to trauma to the trigeminal nerve bundle, which in this case is the mandibular branch (V3), a cascade of events begins, with hyperexcitation and the development of a neurogenic inflammation, facilitating the transmission of nerve impulses toward the central nervous system [4]. Over time, this process, known as peripheral sensitization, can increase neuronal responsiveness and lead to dysfunctions in the descending inhibitory pain system, evolving into a process of central sensitization. This implies an increased presence of sensory inputs toward the somatosensory cortex, which can be interpreted as pain even in the absence of an actual lesion in the periphery [4]. These phenomena can possibly explain the long‐term pain the patient reported in this case, even 1 year after implant placement in addition to the lack of local implant alterations.

The diagnosis of PTNP is usually associated with iatrogenic lesions resulting from more invasive dental procedures, such as implant surgery, for example [3, 14]. However, it is common in the literature to find the presence of PTNP after anesthetic blockade, especially in the inferior alveolar nerve [14], as reported by the patient. Under this hypothesis, the type of anesthetic salt used may have an influence, with articaine being the most frequently associated with this condition; however, mechanical trauma from the needle is also significant in the pathophysiological mechanism [4, 14].

Unfortunately, due to the paucity of studies evaluating potential therapies to control this condition, patients are often left without efficient therapeutic options and are exposed to several professionals and interventions. In the literature, there are reports of the use of pharmacological treatments (analgesics, sedatives, antidepressants, and anticonvulsants), surgical interventions, manual therapies, and even acupuncture [1, 11]. When PTNP is detected in its earliest stages, therapies controlling inflammation may be more resolute, though without robust evidence [2, 11, 14]. After the condition has become chronic, pharmacotherapy with centrally acting medications may be more successful in controlling pain [2]. However, the presence of adverse effects may not be well tolerated by all patients, who may opt for other types of treatment. In the present case reported, there was negligence in detecting the initial traumatic process, followed by a lack of inflammatory control after the iatrogeny. As a result, the patient experienced 12 months of pain, leading to chronification and consequent worsening of the prognosis. Given the patient's personal preference for not using centrally acting medication, acupuncture was chosen.

As a complementary integrative practice, acupuncture is believed to promote a change in the energy in the meridian where the punctured point is related [8]. This therapy has been reported to achieve positive results with a reduction in pain and loss of sensitivity up to 73% [15]. In the present case, the patient showed a significant pain improvement of 88%, associated with a significant reduction in the hyposensitive area. Although still controversial [16], acupuncture has proved to be a promising intervention in the treatment of orofacial pain. The presence of findings with acupuncture no longer performing better than placebo [17, 18] coupled with various studies exhibiting methodological heterogeneity, leads to a conclusion of low reliability [2] for acupuncture as a first‐choice treatment. Nevertheless, given the improvement observed in this case, well‐designed studies on the management of chronic orofacial pain with acupuncture should be encouraged.

The influence of psychological factor is also very much present in the literature, being heavily involved in the pathophysiology of neuropathic pain [11]. It is known that the presence of factors, such as anxiety, depression, and low quality of life, are associated with the onset and maintenance of pain in this condition [1]. This evidence, which aligns with the patient's report of worsening pain and hypoesthesia during stress and anxiety events, reinforces the importance of psychological support as a direct aid in the treatment of this and other orofacial pains [19]. Thus, studies evaluating the efficacy of acupuncture in cases of PTNP should be carried out to establish a better standardization of application, improved patient selection, and the establishment of effective protocols. This should enhance the predictability of results, given the therapeutic potential of this modality, its low invasiveness, and good patient acceptance.

* **Patient Perspective**—“I was very pleased with the acupuncture treatment. Each time I attended an appointment, I experienced a reduction in pain and began to restore sensation in my teeth and face. By the end of the treatment, I no longer felt any pain, and my facial sensation returned to normal. I am extremely grateful and satisfied with the treatment and care provided by the dentist.”

## 3 Conclusion

Therefore, it is important to emphasize that, due to the multifactorial nature of PTNP, the treatment of these cases should not be restricted to a single approach. Although the use of acupuncture was effective in controlling pain and significantly reducing the area of hypoesthesia, further studies are needed to expand the body of evidence on the use of acupuncture to control probable trigeminal neuropathic pain. This case suggests that it may be a promising therapeutic option.

## Conflicts of Interest

The authors declare no conflicts of interest.

## Informed Consent

Informed consent was obtained from the patient.
